# Role of Calprotectin as a Biomarker in Periodontal Disease

**DOI:** 10.1155/2019/3515026

**Published:** 2019-08-21

**Authors:** Lili Wei, Mingwen Liu, Haofei Xiong

**Affiliations:** The State Key Laboratory Breeding Base of Basic Science of Stomatology (Hubei-MOST) & Key Laboratory of Oral Biomedicine Ministry of Education, School & Hospital of Stomatology, Wuhan University, Wuhan, China

## Abstract

Periodontal disease (PD) is a common infectious and inflammatory disease characterised by inflammation of tissues surrounding and supporting the teeth and destruction of the associated alveolar bone, eventually resulting in tooth loss. This disease is caused by periodontopathic bacteria in plaque biofilm and resultant innate and adaptive immune responses in periodontal tissues. Calprotectin (CLP) is a calcium-binding protein of the S-100 protein family and is found to be induced by activated granulocytes, monocytes, and epithelial cells. CLP has been shown to play an important role in numerous inflammatory diseases and disorders. Increasing evidence indicates that CLP is involved in the progression of PD, and its levels may be associated with disease severity and outcome of periodontal treatments. This review will summarise recent studies regarding the presence, regulation, and function of CLP in PD. The findings indicate that CLP may be an effective biomarker for diagnosis and treatment for the PD.

## 1. Introduction

Periodontal disease (PD) is a chronic inflammatory disease caused by infection by oral microorganisms, leading to periodontal tissue damage and alveolar bone destruction and ultimately tooth loss. Although pathogenic plaque biofilms are the primary etiological agents of PD, the disease progression and clinical outcome are determined by host immune responses being modified by microenvironmental and behavioural factors [1, 2]. However, the mechanisms associated with molecular changes in PD remain unclear. Various molecules, including cytokines, hormones, chemokines, and matrix metalloproteinases, and their inhibitors and regulators are involved in disease pathogenesis [3, 4].

Calprotectin (CLP), a member of the S100 family, is a heterodimeric calcium-binding complex protein consisting of two subunits, S100A8 and S100A9 (also known as myeloid-related protein- (MRP-) 8 and MRP-14), which is considered to be the physiologically relevant form and constitutes 5% and 40% of monocytes and neutrophil cytosolic protein contents. CLP is predominantly expressed in myeloid cells, such as neutrophilic granulocytes and inflammatory monocytes/macrophages. The expression of CLP is also inducible in several epithelial cells, microvascular endothelial cells, fibroblasts, keratinocytes, and osteoclasts after activation under certain conditions. However, CLP is absent in resting tissue macrophages and lymphocytes [5–8]. CLP is secreted from myeloid cells during inflammation and exerts its proinflammatory effect on a wide-range type of cells. Recently, CLP has been demonstrated to possibly play a role in the modulation of adaptive host immunity [9]. CLP mainly exists in human plasma, urine, cerebrospinal fluid, faeces, saliva, gingival crevicular fluid (GCF), or synovial fluid and plays an important role in numerous biological functions, including cellular proliferation and differentiation, immunoregulation, oncogenesis, and apoptosis and inflammation [10]. In inflammation, CLP serves as a novel alarm in showing proinflammatory activities mainly induced by activated granulocytes in a calcium-dependent manner; it plays a role by binding to its unique receptors on the cell surface, triggering subsequent signal transductions and inducing leukocyte recruitment and cytokine secretion in inflammatory regions [11, 12].

The effect and underlying mechanisms of CLP in inflammatory and autoimmune diseases have been studied for nearly 20 years. Several inflammatory diseases, including inflammatory bowel disease, chronic bronchitis, rheumatoid arthritis (RA), and psoriasis, are associated with elevated expression levels of CLP. Thus, this protein is a suitable biomarker of inflammatory response and is selected to monitor the response toward anti-inflammation therapy [11, 13]. The role of CLP in pathogenesis of PD has gained considerable attention in recent years. An increasing evidence shows that CLP is involved in PD progression; thus, it might be a candidate biomarker, and its levels are routinely measured during a follow-up of the disease. Here, we will discuss the biologic functions of CLP and review recent studies regarding the collective clinical and *in vitro* evidence of the involvement and role of CPL in PD.

## 2. The Structure, Signalling, and Biological Function of CLP

### 2.1. Structure of CLP

CLP is a 36.5 kDa protein of the S100/calgranulin family. It is a heterodimer consisting of one light (MRP8) and two heavy (MRP14) calcium-binding chains (8 kDa, S100A8/L1L/p8/CP-10, and 14 kDa, S100A9/L1H/p14) [13, 14]. S100A8 and S100A9 share two common EF hand helix-loop-helix motifs bound by a central hinge region. Each subunit can bind two Ca^2+^ ions and other divalent metal ions, such as Zn^2+^ and Mn^2+^. The ionic binding properties of the protein regulate oligomerisation and consequently its functions [15–17]. S100A8 and S100A9 form noncovalently associated heterodimers, which are the most stable form in resting cells and are prevalent under most physiological conditions, especially at low calcium concentration of 0.1 *μ*M. In the presence of high calcium concentrations (10–100 *μ*M), these heterodimers can oligomerise into tetramers, which are transiently formed only during phagocyte activation accompanied by a transient calcium influx. Tetramerisation of S100A8/A9 has been shown to be crucial for the intracellular biological function of proteins, because only tetramers can promote tubulin polymerisation [18–20].

### 2.2. Receptors of CLP

When secreted into extracellular space, CLP accumulates on inflammation sites, interacts with proteins on the cell surface, and triggers special signalling pathways. CLP binds to specific receptors involved in distinct signal transduction; these receptors include Toll-like receptor 4 (TLR4), the receptor for advanced glycation end products (RAGE), and extracellular matrix metalloprotease inducer (EMMPRIN) [19].

TLR4 is a transmembrane signalling protein and important member of TLRs, which belong to a family of pattern recognition receptors. TLR4 has been demonstrated to be capable of sensing pathogen-associated molecular patterns and/or damage-associated molecular patterns (DAMPs) and immediately trigger signal transduction cascade and active inflammatory response. Studies have identified CLP as an important DAMP and an endogenous ligand of TLR4, which exerts double effects on phagocyte homeostasis. In stable circumstances, CLP is involved in cytoskeleton regulation, which can also be released as danger signals when phagocytes are activated [21, 22]. As the main receptor of CLP, TLR4 preferentially binds to heterodimers and activates specific signalling pathways in cells. Multiple downstream signalling cascades result in activation of nuclear factor *κ*B (NF-*κ*B) and upregulation of proinflammatory cytokines and chemokines, which lead to activation of innate immunity and tissue destruction [22, 23]. RAGE belongs to the immunoglobulin (Ig) superfamily and can be expressed in various types of cells, including lymphocytes, monocytes, endothelial cells, neuronal cells, and smooth muscle cells [24]. The binding of RAGE to its special ligands is considered a novel proinflammatory axis and important event in inflammation; RAGE has also been demonstrated to be involved in various inflammatory and autoimmune disorders [25]. Although in vitro binding of RAGE to CLP has been suggested by studies, the relevance of direct receptor-ligand interactions between these molecules in the inflammation process in vivo remains obscure. The gene *Ager* knockout in mouse phagocytes caused no inhibition in response to these cells toward S100A8/S100A9. By contrast, knockout of *Tlr4* completely abolishes the response. Transcriptome analysis has also demonstrated TLR4 as the dominant receptor for S100A8 in human monocytes [21, 26]. EMMPRIN, also known as basigin/CD147, a transmembrane glycoprotein that belongs to the Ig superfamily, mediates the production of various types of MMPs and cytokines and plays important roles in cellular processes, such as cell proliferation and differentiation, apoptosis, angiogenesis, inflammation, and host defence [27]. Studies have provided new knowledge into molecular mechanisms of monocyte/macrophage migration and melanoma cell metastasis mediated by S100A9-EMMPRIN interactions. Notably, S100A9, rather than S100A8 monomer, shows high affinity for EMMPRIN, suggesting that EMMPRIN binds specifically to S100A9. Therefore, the S100A8/SA100A9 heterodimer can also bind to EMMPRIN [28, 29]. However, data confirming the functional relevance of interaction of CLP-EMMPRIN in vivo remain lacking.

### 2.3. Intracellular and Extracellular Biological Functions of CLP

As S100 proteins feature high affinity for binding metal ions, Ca^2+^ buffering was initially suggested as the major intracellular function of CLP in myeloid cells. Intracellular CLP may be involved in Ca^2+^-dependent interaction between the plasma membrane and cytoskeleton. CLP was indicated to interact with actin filaments in nonactivated neutrophils, keratin in epithelial cells, and vimentin in monocytes [11]. In intracellular milieu of high calcium concentrations during cell activation, a functional interaction was found between (S100A8–S100A9)_2_ tetramers and microtubules, promoting tubulin polymerisation and microtubule bundling in resting phagocytes, contrary to phosphorylation of S100A8/S100A9 [18]. This interaction is regulated by p38 mitogen-activated protein kinase (MAPK), signalling the retraction of CLP-mediated tubulin polymerisation and resulting in rapid rearrangement of the microtubule system, which is critical for successful transendothelial migration of phagocytes [20]. In polymorphonucleates (PMNs), intracellular CLP is also critically involved in cytosol tubulin polymerisation and modulation of cytoskeleton, which are important prerequisites for granulocyte migration and may elucidate why CLP can recruit neutrophils during inflammation [30].

Intracellular CLP is also involved in protection against invading pathogens, as epithelial cells expressing CLP are more resistant to bacterial infections than CLP-negative epithelial cells [31]. Mutation in the Ca^2+^-binding loop of S100A9 may lead to complete loss of intracellular antimicrobial activity of CLP, most likely due to the loss of chelating capability of bivalent cations [32]. Furthermore, CLP has been suggested to bind to arachidonic acid in cytosol and transport it to NADPH oxidase complex in the plasma membrane of neutrophils via activation of protein kinase C. By binging on the critical components of NADPH oxidase complex, p67phox and rac-2, CLP induces reactive oxygen species (ROS) production, which is essential for normal functioning of PMNs [33].

In addition to these intracellular functions, CLP is released and acts as an alarm in different inflammatory diseases, showing proinflammatory activity in a wide range of cell types, such as lymphocytes, phagocytes, and endothelial and epithelial cells. Myeloid cells release CLP into extracellular space through passive and active mechanisms. Passive release commonly results from tissue necrosis and destruction or extracellular neutrophil trap formation [11]. By contrast, the specific release of CLP by activated phagocytes during inflammation is an energy-dependent process induced during contact between phagocytes and inflamed endothelium [34]. Neutrophils also release S100A8 and S100A9 and CLP after stimulation with monosodium urate crystals, phorbol myristate acetate, H_2_O_2_, and zymosan; this event depends on ROS production and requires K^+^ exchanges through ATP-sensitive K^+^ channels [35]. After active or passive release by special cells, CLP mediates innate immune response by activating and recruiting immune cells. The presence of elevated levels of CLP in extracellular space has been demonstrated in various inflammatory diseases, indicating that the protein qualifies as a reliable biomarker for inflammation [36–38].

CLP has been demonstrated to exert strong thrombogenic effect on human microvascular endothelial cells (HMECs). CLP stimulates inflammation by increasing the production of different proinflammatory chemokines and expression of adhesion molecules. In addition, CLP disrupts the monolayer integrity of HMECs, leading to loosening of endothelial cell junctions [39]. In recent years, studies have shown that CLP can induce ZO-1 and F-actin disorganisation in human umbilical vein endothelial cells, leading to barrier function disruption and increased endothelial monolayer permeability. The effects depend on activation of p38 MAPK and extracellular signal-regulated kinase (ERK) signalling pathways through TLR4 and RAGE [40]. Kang et al. reported that S100A8/S100A9 homodimers induce MUC5AC production in airway epithelial cells, eliciting both ERK phosphorylation and nuclear translocation of NF-*κ*B in a TLR4-dependent manner. The results suggest that neutrophil-dominant airway inflammation may be associated with mucin hyperproduction [41].

Neutrophils are components of innate host immunity and are rapidly recruited to inflammatory sites, where they can effectively defend against invasive pathogenic bacteria. Treatments with CLP in vitro can stimulate neutrophil chemotaxis, shedding of L-selectin, upregulation, and activation of *β*2 integrin macrophage-1 antigen (Mac-1, *α*M*β*2, and CD11b) on neutrophils and induce adhesion of neutrophils to fibrinogen [42]. Intravenous injection of S100A8/S100A9 or CLP led to rapid recruitment of neutrophils from the bone marrow to the blood. In addition, passive immunisation with anti-S100A9 suppressed the neutrophilia induced by lipopolysaccharide (LPS) injection to the air pouch, confirming the activities of such compound in vivo [43]. Pruenster et al. recently demonstrated that neutrophils rolling on E-selectin present on inflamed endothelium-caused CLP release. Secreted CLP in turn triggered a TLR4-Rap1-GTPase-dependent pathway of rapid *β*2 integrin activation in neutrophils. This extracellular activation cycle reduced rolling velocity of leukocytes and stimulated adhesion, which determined CLP as an important regulator of leukocyte recruitment cascade during inflammation [44].

Considering monocytes, CLP promotes adhesion of monocytes to fibrinogen by increasing the expression level of CD11b on the surface. This increase in adhesion is assumed to contribute to accumulation of monocytes in inflammatory sites [45]. Recently, Wang et al. suggested that human monocytic cell line THP-1, which was activated by CLP, produces C-X-C motif chemokine 10, also known as chemokine *interferon*-*γ*-inducible protein 10 (IP-10). Furthermore, serum levels of IP-10 in patients with blunt trauma were positively correlated with CLP levels and were significantly higher in trauma survivors than in nonsurvivors. These results indicated that CLP might mediate IP-10 expression, which is involved in inflammation induced by injury, in monocytes/macrophages [46].

## 3. The Role of CLP in PD

### 3.1. CLP as Potential Biomarker for PD

CLP is considered to be an important acute phase protein, which is not synthesized in the liver but produced by activated PMNs in circulation and inflammatory tissues. Contrary to cytokines such as TNF-*α*, IL-6, or IL-1*β*, CLP is relatively stable and easy to measure, making it a candidate biomarker for inflammatory diseases [17]. CLP normally exists in human body fluids (e.g., plasma, synovial fluid, saliva, and urine) and faeces, and its level significantly increases in various infectious and inflammatory diseases, including pneumonia, septicaemia, urinary tract infection, rheumatoid arthritis, and inflammatory bowel diseases [14]. Patients with RA have been indicated to present elevated CLP levels in synovial fluid and plasma compared with healthy individuals; this result is closely related to clinical parameters of arthritis inflammation and disease activity [8]. In addition, faecal CLP is an effective marker for diagnosis, classification, treatment, and prevention of inflammatory bowel diseases, such as Crohn's disease and ulcerative colitis [47].

PD is a chronic inflammatory disease caused by oral bacterial infection and results in inflammation in surrounding periodontal tissues and alveolar bone destruction [48]. Various studies have demonstrated the involvement of CLP in the pathology of PD, and altered levels of CLP can be detected in patients with PD. The presence of CLP and its subunits has been observed in human dental calculus, GCF, and saliva [49–51]. A series of cross-sectional studies demonstrated considerably higher levels of CLP in GCF and up to 20-fold higher levels of S100A8 in PD patients than in subjects without PD. Other researches have also suggested that CLP concentrations in GCF are positively correlated with the levels of prostaglandin E2, interleukin- (IL-) 1*β*, collagenase, or aspartate aminotransferase levels and clinical indicators for PD, such as bleeding on probing (BOP), pocket probing depth (PPD), gingival index (GI), and other biomarkers [52–55].

Que et al. quantified CLP and its subunits (S100A8/S100A9) in the GCF during the early phase of experimentally induced gingivitis and investigated variations among inter-/intraindividuals. The findings suggest that CLP levels in the early phase of experimental gingivitis varied between subjects, who were differentiated according to their response patterns [56]. Furthermore, the levels of CLP in GCF from patients with aggressive periodontitis (AgP) and chronic periodontitis (CP) have been suggested to be significantly higher than those of patients with gingivitis and healthy subjects [57]. Zheng et al. indicated that both amount and concentration of CLP in GCF were significantly higher in patients with AgP than those in healthy controls. In addition, the GCF CLP levels were positively correlated with clinical indicators, including BOP, PPD, and clinical attachment loss [58].

In a longitudinal study, Kaner et al. investigated the levels of CLP in GCF before and after nonsurgical therapy of patients with generalised AgP and observed that therapy remarkably decreased levels of CLP, whereas alteration levels were highly related to PPD reduction [59]. In a more complex study of this group, GCF was collected from the initially deepest sites in patients with generalised AgP at 3 months after scaling and root planing therapy, and the samples were collected and determined for CLP levels. Disease activity was defined as the increase in PPD (>0.5 mm) between 3 and 6 months at the same sites. The capability of individual parameters for predicting activity was determined by establishing the *receiver operating characteristic* curve. The findings indicated that the total amount and concentration of CLP showed significant predictive utility. The patients, whose CLP levels were higher than the cut-off values, presented more active sites than the negative patients [60]. These results above indicated that CLP levels in GCF and their alterations were associated with inflammatory circumstances. Moreover, the CLP level is a predictor of disease activity at both site and subject levels for monitoring periodontal therapy in patients with generalised AgP.

Andersen et al. reported that the CLP level in GCF of patients with CP was significantly higher than that of healthy individuals. However, the values showed no difference between healthy and clinically diseased sites in patients. The authors also confirmed that after 3 and 6 months of nonsurgical periodontal treatment, the clinical parameters improved, and the level of CLP decreased significantly in diseased individuals [61]. By contrast, Kajiura et al. investigated CLP concentrations in GCF samples from inflammatory and healthy sites of periodontal pockets in periodontitis patients. The outcomes suggested that CLP levels were significantly higher in inflammatory sites than in healthy sites. In addition, CLP concentrations were positively correlated with GI [62]. In another study by Kajiura et al., the authors indicated that the total amounts of CLP in GCF samples from patients with periodontitis were significantly higher than those of healthy individuals, but CLP concentrations in disease samples were slightly lower. The authors supposed that the differences between concentrations and amounts of CLP were attributed to the increase in GCF volume in inflammatory sites of patients with periodontitis. Furthermore, the authors observed that total CLP amounts in GCF samples from patients with diabetic periodontitis were about twofold higher than those from patients with diabetes mellitus [63]. Notably, the CLP concentrations in GCF from diseased pockets in PD patients were markedly higher (about dozens of times) than that in samples of subjects with other inflammatory disorders. The protein in GCF is thought to be mainly derived from granulocytes and monocytes/macrophages in inflammatory periodontal pockets, entrapping GCF in a specific narrow space and enabling inflammatory cells to produce a large volume and a high level of CLP [52].

Zhou et al. investigated the level alterations of proinflammatory biomarkers, including CLP in saliva, during the early phase of plaque-induced experimental gingivitis in subjects ceasing all oral hygiene measures for 21 days and then resuming the practices for 7 days. The results showed that levels of CLP in saliva gradually increased with plaque accumulation and climaxed on day 21, indicating that CLP levels in saliva could reflect the degree of gingival inflammation [64]. Haririan et al. investigated the levels of CLP in serum and saliva of patients with PD and periodontally healthy individuals. The results indicated that CLP levels were significantly higher in both serum and saliva of patients with PD than those of healthy subjects, but no significant differences were observed between patients with AgP and CP. Furthermore, the levels of CLP in saliva significantly correlated with clinical parameters (e.g., PPD and BOP) and the presence of the periodontopathogen *Treponema denticola*. Lira-Junior et al. indicated that the mean CLP levels in serum were significantly higher (2.06-fold) in generalised AgP patients compared with healthy individuals [65]. However, no significant difference was noted between patients with gingivitis and healthy individuals. Considering saliva, no significant difference in the mean levels of CLP was detected between patients with generalised AgP (or gingivitis) and healthy subjects. Sun et al. discovered that the serum levels of CLP in patients with AgP were significantly higher than those in healthy controls. The authors also suggested higher levels of CLP in the serum of male subjects; the sex difference might be caused by genetic variation in host response caused by S100A8 single-nucleotide polymorphism [66].

Zhan et al. reported that patients with aggressive AgP exhibited a higher status of platelet activation compared with healthy individuals. The levels of CLP in plasma and GCF from patients with aggressive AgP were significantly higher than those of healthy controls. Moreover, the CLP level in GCF was negatively correlated with the platelet large cell ratio and mean platelet volume [67]. In a recent study, Holmström et al. investigated the association between periodontal health and salivary levels of various mediators, including CLP and influence of nondisease variables, on its level in a large cohort of adults. The results suggested that CLP levels were significantly higher in individuals aged >64 years old than in subjects aged 40–64 years old, but no significant differences were observed when compared with the participants aged <40 years old. The CLP levels in saliva were found to be related to periodontal parameters. Mean levels of CLP were 1.37-fold higher in subjects with BOP > 20% in comparison with the ones with BOP ≤ 20% and were 1.35-fold higher in individuals with PPD ≥ 4 mm compared with those with PPD < 4 mm. Furthermore, in this study, the authors observed no significant differences in CLP levels between males and females and between smokers and nonsmokers [68].

Several salivary proteome investigations also demonstrated that S100A8/S100A9 were overexpressed in patients with PD [69–72]. Recent proteomic studies showed that significant differences in expression levels of either or both proteins (depending on the study) might exist in subjects with periodontitis or gingivitis conditions compared with healthy ones. In particular, the levels of both S100A8 and S100A9 in GCF of periodontitis subjects were higher than those of periodontally healthy individuals [73–78].

### 3.2. Cellular Source and Regulation of CLP in PD

As mentioned above, clinical studies demonstrated that CLP levels and their alterations are mainly related to clinical parameters of periodontal inflammation and treatment outcomes of periodontal therapy, suggesting that CLP may be an important inflammatory marker for PD. Here, we will also review the literature regarding the origin and regulation of CLP release from inflammatory cells in PD.

Kido et al. demonstrated that the LPS of *Porphyromonas gingivalis* (P-LPS) increased release of CLP from neutrophils. This induction was inhibited by anti-TLR2 or anti-CD14 antibody but not by anti-TLR4 antibody. The inhibitors of NF-*κ*B or microtubule and microfilament polymerisation significantly suppressed P-LPS-induced CLP release. The findings indicated that P-LPS induced CLP release through the CD14-TLR2-NF-*κ*B signal in human neutrophils and might be dependent on microtubule and microfilament systems [79]. In another study of this group, neutrophils from healthy individuals were stimulated with P-LPS and LPS from *Prevotella intermedia*, *Actinobacillus actinomycetemcomitans*, *Escherichia coli*, and *Fusobacterium nucleatum.* P-LPS could significantly induce the release of CLP in a dose-dependent manner. LPS from *P. intermedia*, *A. actinomycetemcomitans*, *F. nucleatum*, and *E. coli* also increased CLP release from neutrophils [80]. Suryono et al. investigated the expression of CLP in gingival tissue of a periodontitis patient and induction of CLP release from human monocytes by P-LPS and inflammatory cytokines (e.g., *tumor necrosis factor-* (TNF-) *α* or IL-1*β*). The results indicated that CLP was strongly expressed in gingival tissue from periodontitis patients. P-LPS stimulation could induce monocytes to release CLP in a dose-dependent manner, with values increasing to about 2–3 times of the control level, whereas treatment of TNF-*α* and IL-1*β* upregulated CLP release from monocytes to about 2.2 and 1.5 times of the control level, respectively [81]. Suryono et al. further demonstrated that P-LPS, TNF-*α*, or IL-1*β* significantly induced CLP and S100A8/S100A9 mRNA production from human monocytes, and these effects were mediated by activating CCAAT/enhancer binding protein alpha (C/EBP*α*) DNA-binding complex in monocytes [82].

CLP exhibits extensive antimicrobial activity in vitro through zinc chelation and is constitutively expressed in squamous mucosal epithelium (e.g., gingival and vaginal epithelium). In gingival epithelium from periodontally healthy individuals, CLP was found to be expressed in spinous cell layers. During inflammation, CLP localised in the spinous and granular cell layers, and its expression level remarkably increased [83, 84]. Hayashi et al. observed that CLP production and expression of S100A8/S100A9 mRNAs were increased by stimulation with IL-1*α* and calcium through activation of C/EBP*α* DNA-binding complex in normal human gingival keratinocytes. Furthermore, in this previous study, treatment of IL-1*α* and calcium significantly enhanced the CLP level in cell fraction of cultured keratinocytes. However, stimulations showed no effects on the CLP level in the medium fraction (CLP release), whereas the CLP level was substantially lower compared with the cell fraction [85]. This finding was in accordance with the results of Ross et al., who indicated that CLP was expressed constitutively in cultured keratinocytes, whereas S100A8/S100A9 mRNA expression by immortalised keratinocytes was unaffected by exogenous proinflammatory agents IL-1*β* or LPS [86]. Nisapakultorn et al. showed that oral epithelial cells expressing CLP became more resistant to periodontopathic bacteria in PD by suppressing *P. gingivalis* binding and invading to epithelial cells, thus reducing colonisation and persistence of bacteria during periodontal infection and inflammation [31]. In a recent study, Hiroshima et al. investigated the effects of advanced glycation end product (AGE) and P-LPS on CLP expression in the human gingival epithelial cell line OBA-9. The results indicated that AGE acted in synergy with P-LPS to induce RAGE and TLR2 signal transduction and activated MAPK (p38 and c-Jun N-terminal *kinase* (JNK)) and NF-*κ*B signalling pathways, leading to an increased expression of CLP in OBA-9 cells. Furthermore, in the same study, with stimulation of AGE and/or P-LPS, the CLP complex was expressed not only in the cytoplasm but also in the nucleus. This result suggests that CLP might inhibit the growth of accelerated cells mediated by AGE and/or P-LPS by supporting repair of damaged tissues, thus maintaining the function of gingival epithelial barrier to resist infection and inflammation [87]. The findings mentioned above suggest that released CLP may act on periodontopathic bacteria in the presence of neutrophils and monocytes, but in the case of epithelial and keratinocytes, intracellular CLP may act on bacteria invading gingival epithelium.

### 3.3. Biological Effects of CLP on Different Cells in PD

Numerous reports elucidated the functions of CLP in various inflammatory disorders. Previously, CLP has been considered an antimicrobial and antiproliferative protein with calcium-binding property [88]. Recently, CLP and its subunits S100A8/S100A9 have been reported as essential DAMPs recognised by TLR4 and/or RAGE to induce cytokine-like activities [19, 89]. As mentioned above, PD is an infectious disease caused by periodontopathic bacteria during an infectious inflammatory process; CLP may be produced by neutrophils, monocytes, gingival epithelia, and keratinocytes.

Human gingival fibroblasts (HGFs) are one of important cells that regulate cytokine cascades during the development of PD [90, 91]. Nishikawa et al. studied the effects of CLP and its subunit S100A8/S100A9 on the production of inflammatory cytokines in HGFs. The results showed that CLP could significantly increase the production of monocyte chemotactic protein- (MCP-) 1, IL-6, and TNF-*α* in HGFs but not IL-1*β*. S100A9 could significantly increase the production of these four cytokines, whereas CLP and S100A9 showed no significant difference in MCP-1 and IL-6 productions. Compared with CLP and S100A9, S100A8 exerted a lower inductive effect on MCP-1, IL-6, and TNF-*α* and presented negligible pathophysiological significance. In this study, HGFs constructively expressed TLR4 rather than RAGE mRNA, suggesting that TLR4 may be a major target receptor of CLP in HGFs. In addition, CLP significantly increased the production of inflammatory cytokines by activating the signalling pathways of NF-*κ*B and MAPKs (ERK, JNK, and p38 MAPK), and the production was markedly inhibited on HGFs by treatment with NF-*κ*B, MAPKs, and TLR4 inhibitors [23]. In another study, Gao et al. investigated the proinflammatory activity of CLP and its subunits on HGFs and functional receptors and signalling pathways involved in this progression. The results indicated that CLP and S100A9 significantly increased the expressions of IL-6 and IL-8 in HGFs and suggested that CLP exerts proinflammatory activity on HGFs through subunit S100A9 and TLR4-mediated NF-*κ*B and MAPK signalling pathways [92]. From these results, it can be concluded that CLP could induce cytokine production in HGFs via TLR4 signal, which involves NF-*κ*B and MAPK activation, thus playing an important role in the pathology of PD.

Zheng et al. studied the effects of CLP and its subunits S100A8/S100A9 on periodontal ligament cells (PDLCs). The results indicated that CLP promoted apoptosis of PDLCs, whereas S100A8 and S100A9 individually showed minimal effect on cell apoptosis. CLP and S100A9 increased the activation of NF-*κ*B by promoting nuclear translocation of p65 in PDLCs and then induced the production of cytokines, including IL-6, IL-8, *cyclooxygenase*-2, and TNF-*α*. NF-*κ*B inhibition partially reversed the upregulation of CLP and S100A9-induced proinflammatory cytokines. S100A9, rather than S100A8, mainly caused the proinflammatory effect of CLP. These findings suggest that CLP promotes cellular apoptosis and inflammation in PDLCs via S100A9, consistent with the results of Gao et al. They demonstrated that S100A9 promoted proinflammatory activity in PDLCs via TLR4-mediated NF-*κ*B and MAPK signalling pathways [58, 93].

Recently, Kajiura et al. reported that CLP could induce significant production of soluble form of IL-6 receptor (sIL-6R) in THP-1 macrophages. IL-6 is a critical proinflammatory cytokine that elicits multiple functions in the pathogenesis of PD, and sIL-6R presents agonistic activities for IL-6 in required cells [62]. Considering that HGFs show no expression of surface IL-6R in response to IL-6 [94], CLP-induced IL-6 in HGFs would respond to themselves by an autocrine mechanism in the presence of sIL-6R released from macrophages. That is to say, CLP induces the production of IL-6 and sIL-6R in HGFs and macrophages, and such event may play a role in PD progression through crosstalk between fibroblasts and macrophages [95].

TLR inhibition may be helpful in the treatment of various inflammatory disorders, such as RA and PD [96, 97]. Many studies have been carried out to investigate how cytokines are released from fibroblasts (e.g., HGFs and PDLCs) and their biological effects in inflammatory periodontal lesions. Based on the above results, it can be indicated that fibroblast-mediated inflammation may also be regulated by CLP via TLR4 signalling, suggesting one of the potential mechanisms for PD pathogenesis, which could be an attractive therapeutic target for treating the disease.

## 4. Concluding Remarks

This article aims to provide readers with better understanding of the role of CLP in PD pathogenesis. The findings suggest the upregulation of CLP in human PD and the relationship between CLP levels and disease severity. CLP in particular is an effective biomarker for monitoring disease activity and outcomes of periodontal treatments. Owing to its local pattern of expression and release, CLP induces not only bacteriostatic effects but also cytokine-like effects in the local environment, promoting inflammation and periodontal tissue damage. That is to say, CLP may exert protective and destructive effects during the onset and development of PD. It may play a critical role in neutrophil activation and recruitment and thus prevent against infection-stimulated inflammatory tissue destruction in PD. In addition, CLP may also be one of the factors responsible for stimulating the production of inflammatory mediators, with most of them destabilizing the local immune homeostasis by promoting periodontal tissue destruction and bone resorption. However, the exact role of CLP in PD is still equivocal; it is uncertain whether the overall effect of CLP is to combat ongoing infection or contribute to tissue and bone destruction by promoting inflammatory response. Further research is still required on the role of CLP, especially the underlying mechanisms that drive CLP response and regulate inflammation-induced tissue destruction, to maximize host protective effects and minimize host-deleterious effects. A greater understanding of these mechanisms may help in the development of new therapeutic strategies for inflammatory PD.
